# Clinical course of IPF in Italian patients during 12 months of observation: results from the FIBRONET observational study

**DOI:** 10.1186/s12931-021-01643-w

**Published:** 2021-02-24

**Authors:** V. Poletti, C. Vancheri, C. Albera, S. Harari, A. Pesci, R. R. Metella, B. Campolo, G. Crespi, S. Rizzoli, the FIBRONET study group, C. Vancheri, S. Tomassetti, S. Harari, A. Pesci, C. Albera, P. Rottoli, M. Bocchino, A. A. Stanziola, F. Luppi, A. Sebastiani, D. Lacedonia, P. Vitulo, L. Tavanti, A. Vianello, M. Saetta, S. Marinari, P. Pirina, S. Valente, T. Oggionni, S. Gasparini

**Affiliations:** 1 Department of Diseases of the Thorax, Ospedale GB Morgagni, Forlì, Italy; 2 Department of Respiratory Diseases & Allergy, Aarhus University Hospital, Aarhus, Denmark; 3 Regional Referral Centre for Rare Lung Diseases—University Hospital “Policlinico G. Rodolico”, Department of Clinical and Experimental Medicine, University of Catania, Catania, Italy; 4 S.C. Pneumologia U., A.O.U. Città Della Scienza E Della Salute (Molinette), University of Torino, Torino, Italy; 5 Department of Clinical Sciences and Community Health, University of Milan, Milan, Italy; 6 Department of Medicine, Ospedale San Giuseppe MultiMedica IRCCS, Milan, Italy; 7 Ospedale San Gerardo, ASST Monza, Monza, Italy; 8 Dipartimento di Scienze Mediche Chirurgiche e Neuroscienze, Università degli Studi di Siena, Siena, Italy; 9 Boehringer Ingelheim, Milan, Italy; 10 MediNeos Observational Research, Modena, Italy

**Keywords:** Idiopathic pulmonary fibrosis, Real-world, Observational, Italy, Antifibrotic therapy, Nintedanib, Pirfenidone, Lung function

## Abstract

**Background:**

FIBRONET was an observational, multicentre, prospective cohort study investigating the baseline characteristics, clinical course of disease and use of antifibrotic treatment in Italian patients with idiopathic pulmonary fibrosis (IPF).

**Methods:**

Patients aged ≥ 40 years diagnosed with IPF within the previous 3 months at 20 Italian centres were consecutively enrolled and followed up for 12 months, with evaluations at 3, 6, 9 and 12 months. The primary objective was to describe the clinical course of IPF over 12 months of follow-up, including changes in lung function measured by % predicted forced vital capacity (FVC% predicted).

**Results:**

209 patients (82.3% male, mean age 69.54 ± 7.43 years) were enrolled. Mean FVC% predicted was relatively preserved at baseline (80.01%). The mean time between IPF diagnosis and initiation of antifibrotic therapy was 6.38 weeks; 72.3% of patients received antifibrotic therapy within the first 3 months of follow-up, and 83.9% within 12 months of follow-up. Mean FVC% predicted was 80.0% at baseline and 82.2% at 12 months, and 47.4% of patients remained stable (i.e. had no disease progression) in terms of FVC% predicted during the study.

**Conclusions:**

FIBRONET is the first prospective, real-life, observational study of patients with IPF in Italy. The short time between diagnosis and initiation of antifibrotic therapy, and the stable lung function between baseline and 12 months, suggest that early diagnosis and prompt initiation of antifibrotic therapy may preserve lung function in patients with IPF.

*Trial registration:* NCT02803580

## Background

The interstitial lung diseases (ILDs), a heterogeneous group of over 200 distinct diseases, include a subgroup of diseases known as the idiopathic interstitial pneumonias (IIPs) [1, 2]. The most common IIP is idiopathic pulmonary fibrosis (IPF) [1], a progressive, and ultimately fatal, disease characterised by progressive fibrosis of the lung parenchyma and subsequent decline in lung function, and defined by a usual interstitial pattern on high-resolution computed tomography (HRCT) [3–5]. IPF shows a variable rate of progression and the disease course may include acute, life-threatening exacerbations [6, 7]. The disease is more frequent in men than in women and is usually diagnosed in people aged over 50 years, particularly in those with a history of smoking [3]. Prior to the availability of antifibrotic treatment, the prognosis for IPF was poor (3–6 years) [8–10].

Diagnosing IPF in clinical practice can be challenging, as symptoms often appear similar to those of more common respiratory diseases, such as chronic obstructive pulmonary disease. This contributes to a delay in diagnosis, which is usually made 6–24 months after initial symptoms [11–13], but can be made even later (> 3 years) [14]. In 2011, simplified and updated criteria for the diagnosis and management of IPF were published by the American Thoracic Society (ATS), European Respiratory Society (ERS), Japanese Respiratory Society (JRS), and Latin American Thoracic Society (ALAT); this document was updated again in 2018 [3, 15]. Early diagnosis is important as it enables earlier treatment and, potentially, improvement of long-term clinical outcomes. There is currently no therapy that reverses or cures the lung damage associated with IPF; however, two antifibrotic drugs (pirfenidone [Roche] and nintedanib [Boehringer Ingelheim]) are licensed for the treatment of IPF, both of which can slow the decline in lung function [16, 17]. Despite the availability of these drugs, many patients diagnosed with IPF are not treated with an antifibrotic. According to one survey of 290 respiratory physicians in Europe, 40% of patients with a confirmed diagnosis of IPF in Italy, Spain, France, Germany and the UK do not receive treatment with an approved antifibrotic [18].

The epidemiology of IPF in Italy has not been thoroughly investigated. Two regional studies estimated the annual incidence of IPF to be between 2.5 and 5.3 cases per 100,000 person-years in Lombardy, and between 7.5 and 25.6 per 100,000 person-years in Lazio [19, 20]. In another national study of 1,104,037 patients with IPF in the Italian primary care setting between 2002 and 2017, the incidence of IPF based on health database records was estimated to be 1.25–3.77 [21]. However, epidemiological studies of IPF are widely acknowledged as challenging due to changes in IPF diagnostic criteria over time and modifications to the IPF coding systems used in administrative databases (a common source of epidemiological data), as well as differences in study design, methodology and study populations in clinical trials [22].

Long-term data on the natural course of IPF in Italy are scarce, and there is limited information on patient characteristics and disease management. However, a growing body of real-world evidence from Italy suggests that pirfenidone and nintedanib can attenuate the decline in lung function in patients with IPF [23, 24], and data from other real-world studies in Europe and Australia suggest that antifibrotic treatment may prolong survival [10, 25, 26]. Integrating evidence from randomised controlled trials with real-life evidence has become increasingly important for the respiratory physician, since real-life studies are not limited by strict selection criteria and therefore approximate the general patient population more accurately [27]. Adding to the existing body of real-life evidence in Italy, the primary objective of this study was to describe the baseline characteristics, the clinical course of the disease (in terms of changes in lung function, including forced vital capacity [FVC]% predicted) and the use of antifibrotic treatment in a group of Italian patients with IPF, during 12 months of observation.

## Methods

### Study design

This was an Italian, observational, multicentre, prospective cohort study enrolling approximately 200 patients meeting the inclusion/exclusion criteria described below over 18 months. Patients were followed up for 1 year, undergoing three follow-up evaluations after 3 (± 1.5), 6 (± 1.5) and 9 (± 1.5) months (as per current clinical practice in Italy for the management of patients with IPF), and a final follow-up visit at 12 (± 1.5) months. In total, 20 pulmonary centres, managing the majority of patients with IPF in Italy, were involved in the study.

### Inclusion and exclusion criteria

Patients were included if they were aged ≥ 40 years, with a diagnosis of IPF confirmed by a physician during the previous 3 months (based on 2011 ATS/ERS/JRS/ALAT guidelines, as this study was started before the 2018 update) [3] and an assessment of IPF based on HRCT, or HRCT and surgical lung biopsy if required and available. Patients were excluded if they were participants in other clinical trials or other IPF or ILD registries, were scheduled to receive a lung transplant within the next 6 months, or were pregnant or breastfeeding. The protocol was approved in July 2015 by an institutional review board/ethics committee (107/2015/PO del registry EC), and written informed consent was obtained for all patients.

### Outcomes

The primary objective was to describe the clinical course of IPF in a group of patients in Italy by assessing symptoms, lung function and exercise tolerance during 12 months of observation. Of these, change in FVC% predicted as a measure of lung function was our primary endpoint of interest. Key secondary endpoints included patient characteristics at enrolment in terms of key socio-demographic data, IPF risk factors, comorbidities, acute IPF exacerbations, and hospitalisations and other medical visits. Health-related quality of life was assessed by the St. George’s Respiratory Questionnaire (SGRQ), originally developed to measure the impact of obstructive airway disease on overall health, daily life and perceived well-being [28]. The SGRQ scale ranges from 0–100, with higher scores indicating greater limitations. In addition, treatment-related data were collected (time from diagnosis to treatment initiation, as well as percentage of patients receiving antifibrotic therapy at each follow-up time point).

### Statistics

Missing data for patients lost to follow-up were not imputed for the primary analysis, although two additional sensitivity analyses were performed using a ‘last observation carried forward’ approach. In the first analysis set, evaluable patients were those with FVC% predicted available at baseline and who underwent pulmonary function tests (PFTs) at one or more follow-up visit. In the second analysis set, evaluable patients were those with FVC% predicted available at baseline and who underwent PFTs at 6-, 9- or 12-month follow-up.

Descriptive analyses included means, medians, quantiles, proportions and contingency tables, according to the nature of the variables. As a dispersion measurement, the standard deviation (SD) and the interquartile range were calculated. Statistical analyses were performed on evaluable patients, defined as those with known gender who fulfilled inclusion/exclusion criteria and completed at least one of the following evaluations at enrolment: IPF symptoms, lung function and 6-min walk test. For analysis of follow-up data, only patients who completed Visit 2 (3 months), Visit 3 (6 months), Visit 4 (9 months) and Visit 5 (12 months) were considered. Patients needing lung transplantation were not excluded from the analyses or analysed separately.

## Results

### Patient characteristics

Patients were enrolled between 30 November 2015 and 6 April 2017. In total, 209 patients were evaluable for analysis at enrolment. IPF was diagnosed using HRCT in all patients (100.0%). Other diagnostic procedures included spirometry (27.8%), bronchoalveolar lavage (19.1%), cryobiopsy (8.1%), surgical lung biopsy (6.7%) and transbronchial biopsy (5.3%). Nineteen of the 20 participating centres had at least one patient evaluable at the 12-month follow-up visit. A high proportion of patients were evaluable at each time point: 191 (91.4%) at 3 months, 172 (82.3%) at 6 months, 161 (77.0%) at 9 months, and 174 (83.3%) at 12 months (Table 1). During the 12-month observation period, 36 patients (17.2%) withdrew from the study. Of these, there were 13 (6.2%) deaths, 19 (9.1%) lost to follow-up, three (1.4%) who withdrew consent, one (0.5%) who was included in a clinical trial and one (0.5%) who withdrew for other reasons (a patient could withdraw from the study for more than one reason).

**Table 1:** Patient disposition

	n (%)
Enrolled	209 (100)
Evaluable at enrolment	209 (100)
Performed V2 (3 ± 1.5 months)	191 (91.4)
Performed V3 (6 ± 1.5 months)	172 (82.3)
Performed V4 (9 ± 1.5 months)	161 (77.0)
Performed V5 (12 ± 1.5 months)	174 (83.3)
Withdrawn patients	36 (17.2)
Inclusion in clinical trial	1 (0.5)
Consent withdrawal (from patient or legally accepted representative)	3 (1.4)
Lost to follow-up	19 (9.1)
Death	13 (6.2)
Other cause of discontinuation	1 (0.5)

The mean (± SD) number of months between baseline and follow-up visits was 3.24 (± 0.58) months for V2 follow-up, 6.26 (± 0.58) months for V3 follow-up, 9.16 (± 0.61) months for V4 follow-up, and 12.32 (± 0.66) months for V5 follow-up

*SD* standard deviation, *V* Visit

Most patients (82.8%) were male. The mean age at enrolment (± 1 SD) was 69.54 (± 7.43) years, and all patients were Caucasian/white. The mean (± SD) weight (kg) was 77.95 (± 13.18) and the mean (± SD) height (cm) was 166.88 (± 8.36). According to the body mass index (BMI), 50 (29.9%) participants were classified as obese (BMI ≥ 30), 81 (48.5%) as overweight (BMI 25–29.9), 34 (20.4%) as normal weight (BMI 18.5–24.9) and 2 (1.2%) as underweight (BMI < 18.5). At baseline, nine patients (4.3%) were receiving nintedanib and 24 (11.5%) pirfenidone.

The most frequently reported comorbidities were arterial hypertension (n = 103, 49.3%), gastroesophageal reflux disease (n = 49, 23.4%), diabetes mellitus (n = 45, 21.5%), atherothrombotic disease including coronary heart disease (n = 27, 12.9%), and benign prostatic hypertrophy (n = 25, 12.0%) (Table 2). The majority of patients were former smokers (n = 138, 66.0%) and a small proportion (n = 9, 4.3%) were current smokers. The median (25th–75th percentile) estimated amount of tobacco consumed by former and current smokers was 30 (14–40) packs per year for a median (25th–75th percentile) of 30 (20–40) years. Seventy patients (34.0%) reported previous exposure to environmental risk factors (e.g. metal dust, asbestos) (Table 3). Two patients (1.0%) had been exposed to amiodarone, and 25 (12.0%) had a family history of pulmonary fibrosis.

**Table 2:** Comorbidities at enrolment

	n (%)
N of patients with at least one comorbidity	171 (81.8)
Anxiety/depression	12 (5.7)
Arterial hypertension	103 (49.3)
Atherothrombotic disease, including coronary heart disease	27 (12.9)
Atrial fibrillation	9 (4.3)
Benign prostatic hypertrophy	25 (12.0)
Cerebrovascular disease (carotid stenosis, stroke)	13 (6.2)
Deep venous thrombosis (DVT)	1 (0.5)
Diabetes mellitus	45 (21.5)
Emphysema	8 (3.8)
Gastroesophageal reflux disease	49 (23.4)
Hypercholesterolaemia	17 (8.1)
Lung cancer	1 (0.5)
Peripheral arterial disease (symptomatic or ankle brachial index < 0.8)	2 (1.0)
Pulmonary hypertension	6 (2.9)
Renal insufficiency	5 (2.4)
Other	80 (38.3)
Previous myocardial infarction	23 (11.0)

Percentages calculated out of total number of evaluable patients. A patient could have more than one comorbidity

**Table 3:** IPF risk factors and family history for pulmonary fibrosis

	N = 209
Exposure to environmental risk factors	
No	136 (65.1)
Yes	70 (33.5)
Not known	3 (1.4)
Metal dust	27 (12.9)
Wood dust	9 (4.3)
Solvents	15 (7.2)
Oils	6 (2.9)
Asbestos	17 (8.1)
Quartz dust	2 (1.0)
Farming	2 (1.0)
Livestock dust	1 (0.5)
Vegetable dust	4 (1.9)
Other	12 (5.7)
Exposure to amiodarone	
No	207 (99.0)
Yes	2 (1.0)
Family history	
No	183 (87.6)
Yes	25 (12.0)
Not known	1 (0.5)

*IPF* idiopathic pulmonary fibrosis

The mean (± SD) number of months from first IPF diagnosis to inclusion in the study was 1.01 (± 1.05), and the mean (± SD) number of years from first IPF symptoms to inclusion in the study was 2.14 (± 2.45). At first IPF diagnosis, the mean (± SD) age was 69.46 (± 7.43) years.

## Results: primary endpoints

### IPF symptoms and exercise tolerance

At baseline, 184/209 evaluable patients (88.0%) had symptoms of IPF. The most frequent symptom at baseline was cough (59.8%), followed by fatigue (54.1%) and dyspnoea (18.2%). At 12 months (n = 174), the frequencies of these symptoms were 30.5%, 32.2% and 4.6%, respectively. Dizziness and chest pain (evaluated separately) were reported in < 5% of patients at baseline and 12 months. The mean (± SD) 6-min walk distance was 395.70 (± 121.70) metres at baseline, and 411.70 (± 108.90) metres at 12 months. No sensitivity analyses were applied to the longitudinal data for either symptoms or exercise tolerance.

### Lung function

**Fig. 1:**
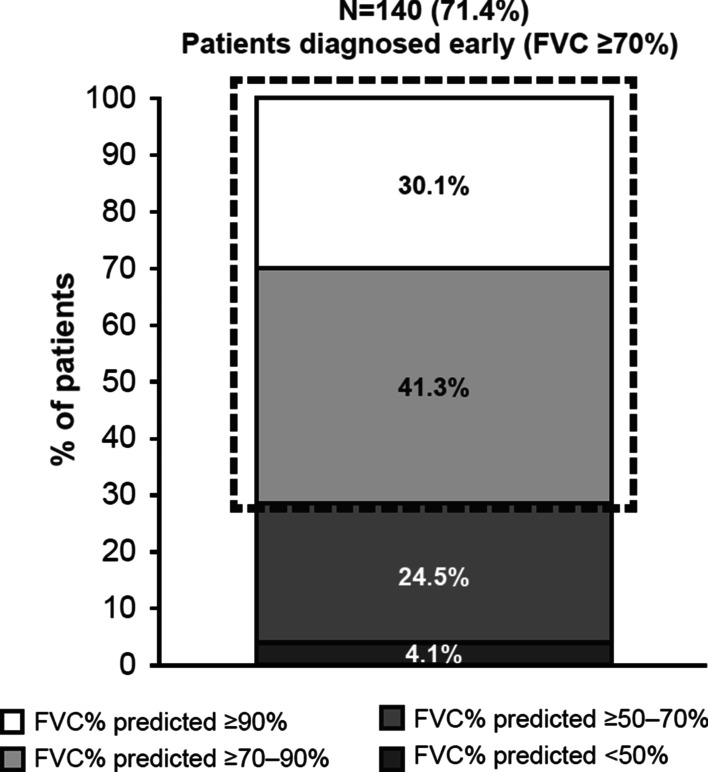
FVC% predicted at baseline. Percentages calculated from the total number of evaluable patients with available FVC predicted at baseline (n = 196). *FVC* forced vital capacity

**Fig. 2:**
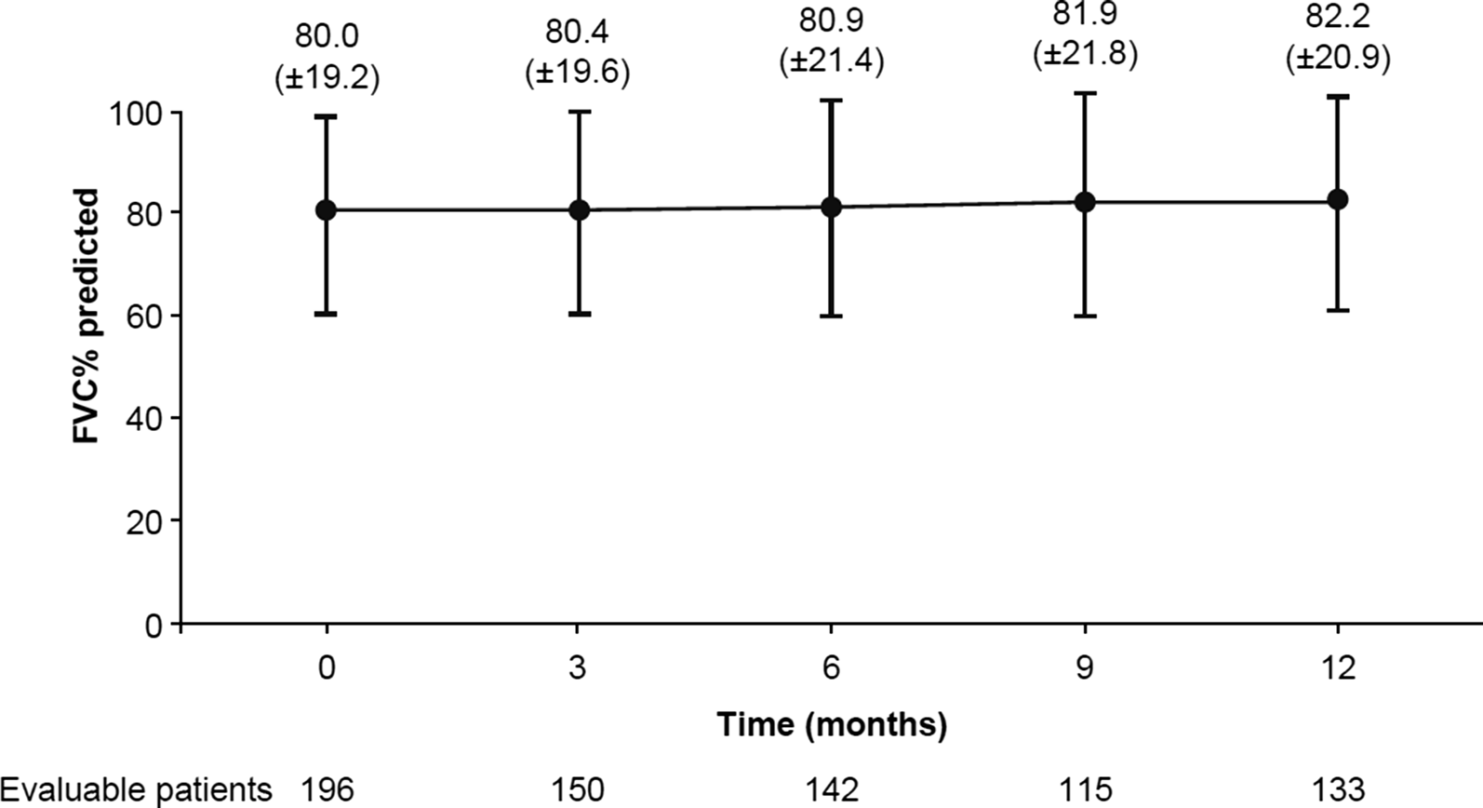
Mean change in FVC% predicted (± SD) during 12 months of observation. *FVC* forced vital capacity, *SD* standard deviation

At baseline, 140/196 evaluable patients (71.4%) had FVC predicted ≥ 70% (Fig. 1) and 59/196 (30.1%) had FVC predicted ≥ 90% (suggesting very early diagnosis). In the whole group, FVC% predicted did not decline over time: the mean (± SD) FVC% predicted was 80.01% (± 19.23) at baseline and 82.17% (± 20.93) at 12-month follow-up (Fig. 2). Considering the relative change of FVC% predicted at 12 months versus baseline, 52.6% of evaluable patients (70/133) were classified as ‘decliners’ (> 0% decrease in FVC% predicted). Of these, 24.1% decreased by 0– < 5%, 12.0% decreased by 5– < 10%, and 16.5% decreased by ≥ 10%. The remaining patients (63/133; 47.4%) were classified as ‘non-decliners’ (> 0% increase in FVC% predicted). Of these, 15.0% increased by 0– < 5%, 15.0% increased by 5– < 10%, and 17.3% increased by ≥ 10% (Table 4). In the first sensitivity analysis (imputation of missing values for patients with values at baseline and any follow-up), mean FVC% predicted (± SD) was 80.18% (± 20.41) at 12-month follow-up (for 183 evaluable patients). In the second sensitivity analysis (imputation of missing values for patients with values at baseline and 6-, 9- or 12-month follow-up), mean FVC% predicted (± SD) was 80.12% (± 20.31) at 12-month follow-up (for 173 evaluable patients) (Additional file 1: Table S1). The sensitivity analyses had a marginal impact on the overall results: the proportion of decliners ranged between 52.0% and 53.0%, very close to the proportion observed in the primary analysis (52.6%). Along with spirometry measurements, diffusing capacity of the lung for carbon monoxide (DL_CO_) remained stable during observation. The mean (± SD) DL_CO_% predicted at baseline and 3, 6, 9 and 12 months’ follow-up was 51.68% (± 13.52), 51.27% (± 14.75), 49.71% (± 13.23), 50.27% (± 16.58) and 50.91% (± 15.65) respectively.

**Table 4:** FVC% predicted relative change from baseline to 12 months

	N = 133
Decliners (decrease in FVC% predicted)	70 (52.6%)
0– < 5%	32 (24.1%)
5– < 10%	16 (12.0%)
10– < 15%	8 (6.0%)
≥ 15%	14 (10.5%)
Non-decliners (increase in FVC% predicted)	63 (47.4%)
0– < 5%	20 (15.0%)
5– < 10%	20 (15.0%)
≥ 10%	23 (17.3%)

*FVC* forced vital capacity

## Results: secondary endpoints

### Use of antifibrotic and other therapy during study

A total of 33 patients (15.8%) were receiving antifibrotic treatment at baseline (within the 3 months prior to enrolment), and the overall proportion of treated patients increased during the observation period: 72.3% (n = 138) at 3 months, 80.8% (n = 139) at 6 months, and 83.9% (n = 146) at 12 months. The majority of evaluable patients began antifibrotic treatment early in the study (within 3 months prior to enrolment, or within the first 3 months of the study), with only eight patients initiating treatment later and 28/146 evaluable patients (16.1%) receiving no antifibrotic therapy at 1 year. The mean time from diagnosis to initiation of treatment was 6.38 weeks.

Non-antifibrotic therapy was primarily long-term oxygen therapy but was not frequently used: 3.3%, 8.4%, 8.1% and 8.6% at baseline, 3, 6, and 12 months, respectively. No patients were listed for lung transplantation at baseline, and the transplantation rate remained low throughout the study: 2.6% (n = 5) at 3 months, 1.2% (n = 2) at 6 months, and 0.6% (n = 1) at 12 months. Adherence to antifibrotic medications (according to 4-item Morisky Medication Adherence Scale score) was good, with no relevant changes over time: mean (± SD) 3.70 (± 0.65) at baseline, 3.65 (± 0.74) at 6 months, and 3.56 (± 0.75) at 12 months.

### Health-related quality of life

The median (25th–75th percentile) SGRQ total score at baseline was 39.82 (23.40–56.49), and was worst in the ‘symptoms’ (42.26 [27.29–57.98]) and ‘activities’ (54.39 [35.80–72.89]) subcategories, and highest in the ‘impacts on daily life’ (28.16 [12.26–47.09]) subcategory (Additional file 1: Figure S1). After 12 months of follow-up, SGRQ scores were similar to baseline in all subcategories (Additional file 1: Table S2). No sensitivity analyses were performed on these data.

### Hospitalisation, emergency room admission and other medical visits

Thirteen patients (6.2%) had at least one hospitalisation, with a total of 14 hospitalisations recorded. Ten patients (4.8%) had a total of 15 admissions to the emergency room. 29.7% of patients (62/209) had at least one medical visit, the most frequent of which were visits to the general practitioner (GP) clinic (n = 48, 23.0%), followed by the pulmonologist clinic (n = 23, 11.0%) (Additional file 1: Figure S2). Considering patients who visited a clinic, the mean (± SD) number of GP and pulmonologist visits per patient was 1.60 (± 0.82) and 1.61 (± 0.92), respectively. During the study, 121 patients (57.9%) underwent laboratory examinations at pre-specified follow-up visits, mainly due to the required surveillance for antifibrotic therapy or to monitor IPF progression (especially liver function tests, blood count tests, HRCT and unscheduled spirometry).

### Acute IPF exacerbations and adverse events

In total, 15 patients (7.2% of those evaluable at enrolment) had at least one exacerbation during the observation period. There were 18 exacerbations reported (seven mild, seven moderate and four severe, according to clinical judgement). Five patients (2.4%) had one mild exacerbation, seven patients (3.3%) had one moderate exacerbation, two patients (1.0%) had one severe exacerbation, one patient (0.5%) had two severe exacerbations and one patient (0.5%) had two mild exacerbations. The 1-year Kaplan–Meier estimate of the probability of an exacerbation was 6.5%.

Safety was not one of the primary or secondary objectives of this study. However, 17 serious adverse events were recorded in total, 11 of which were fatal (Additional file 1: Table S3). In addition to these 11 deaths, 2 further deaths occurred during the observation period. No sensitivity analyses were performed on these data.

## Discussion

The main socio-demographic and clinical characteristics of Italian patients in this real-world study are consistent with those previously described in the literature (Additional file 1: Table S4). At baseline, mean FVC% predicted was relatively preserved in FIBRONET (80.01%). This is similar to another real-world study of 128 Italian patients with IPF, in which mean FVC% predicted was 75.0% at baseline and 74.0% after 1 year of treatment with pirfenidone [28], and slightly higher than in a study of 41 Italian patients with advanced IPF (60.3% at baseline and 58.0% after 6 months) [24]. Almost half of the patients in FIBRONET (47.4%) remained stable (i.e. had no disease progression) in terms of FVC% predicted during 12 months of observation.

These results are consistent with findings from previous clinical trials in IPF. In a post hoc analysis of FVC (mL) change from baseline at Week 52 in the INPULSIS® and INPULSIS®-ON trials, 191 patients (36.8%) in the nintedanib group and 62 patients (18.0%) in the placebo group showed no decline in FVC [29]. In the sample of 624 patients with IPF who met the criteria for enrolment in the CAPACITY® or ASCEND® trials and were assigned to placebo, 91% of patients showed either no decline or a < 10% decline in the first 6 months of the study [30]. Another observational study conducted in 20 ILD expert centres in Germany (INSIGHTS-IPF) also found that FVC was relatively stable over the first 12 months of antifibrotic treatment, in line with our findings [25]. Although several clinical trials and real-world experiences show that a proportion of patients has either no decline or < 10% decline in FVC during 12 months of observation, it is not appropriate to describe such patients as having ‘stable disease’ since IPF is by nature a progressive disease. The SGRQ total score at baseline (39.82) in our study was slightly lower (suggesting less of an impact on quality of life) than the scores in the Australian registry (44.3) and INSIGHT registry (47.7) [26]. The changes in SGRQ total score over approximately 1 year were small in both the Australian and INSIGHT registry [31, 32]. At 12 months, we observed fewer patients reporting cough, fatigue and dyspnoea, although it is unknown if this was influenced by patient drop-outs or related to the treatment.

### Antifibrotic therapy

An earlier diagnosis of IPF allows earlier treatment and, potentially, improvement of long-term clinical outcomes. Despite the available scientific data from clinical trials, post hoc analyses, long-term safety studies and real-world experiences, the question of when to start and when to stop treatment with antifibrotics is still under debate. In IPF, particularly when the disease is diagnosed at an early stage, ‘wait and watch’ is a common approach. This is largely due to the lack of awareness of both patients and physicians regarding the progression of the disease and its prognosis. In FIBRONET, the mean (± SD) number of years between the first symptoms of IPF and study enrolment was 2.14 (± 2.45), whereas on average, only 6.38 weeks elapsed between IPF diagnosis and initiation of antifibrotic therapy. We speculate that the short time between diagnosis and initiation of antifibrotic therapy, combined with relatively preserved lung function at baseline (FVC 80.01%), is a possible explanation for the relatively stable lung function observed over 52 weeks in our study (47.4% with marginal or no decline in FVC% predicted after 12 months of observation).

The proportion of patients receiving antifibrotic treatment increased over the course of the study (15.8% at baseline, 72.3% at 3 months, 80.8% at 6 months, and 83.9% at 12 months). The overall proportion of patients receiving antifibrotic treatment in this study is much higher compared with Finnish and Swedish IPF registry studies conducted between 2014 and 2016, in which the percentage of patients receiving antifibrotic therapy over a 3-year period was 29.6% and 69.4%, respectively [33]. A survey of French pulmonologists treating patients with IPF in 2014 found 31% of physicians treated patients with antifibrotics [34]. The differences may be explained by different types of centres participating in registry activities, i.e. expert centres in the management of ILD in the FIBRONET study, compared with perhaps a broader range of centres participating in the Finnish and Swedish registries. Differences in reimbursement for antifibrotic drugs between countries may also explain some of these differences (see Pesonen et al. [33]). Lastly, Italy has a relatively well-established and strong ILD community within a universal healthcare system, which may influence prescription rates.

### Limitations

FIBRONET was a descriptive, observational study in which patients were prospectively followed for 1 year, so neither causality nor treatment association was evaluated, and possible confounders were not assessed. The number of patients enrolled into our study is not as large as some other registries, but we believe it provides a useful representation of the Italian population. One limitation of our study is the high number of patients lost to follow-up (76 patients [36.4%] with missing FVC data at 12-month follow-up), which may have biased the results, since patients with greater disease progression may not have been healthy enough to attend their follow-up visits. In order to minimise selection bias, patient sampling was based on consecutive enrolment, and every effort was made to select sites across a variety of geographic regions. The patients in this study were all treated at specialist centres, and the results may therefore not be comparable with other real-world studies involving non-specialist centres. However, since the specialist centres that were selected treat the majority of patients with IPF in Italy, our study does reflect the healthcare setting for most patients in that country. Lastly, we did not collect data on treatments used by patients other than nintedanib and pirfenidone during the observation period.

## Conclusions

FIBRONET is one of the largest prospective real-world studies of Italian patients with IPF and is unique in that it was designed to prospectively describe the clinical course of IPF in terms of changes in lung function in an Italian real-world context. The results of this study suggest that early diagnosis of IPF, enabling early initiation of antifibrotic therapy, may be associated with preserved lung function in patients with IPF. These results add some relevant data to the body of observational, real-life, long-term data on the natural course of IPF in Italy, which currently are limited.

## Supplementary Information

**Additional file 1.** Additional tables and figures.

## Data Availability

To ensure independent interpretation of clinical study results, Boehringer Ingelheim grants all external authors access to all relevant material, including participant-level clinical study data, and relevant material as needed by them to fulfil their role and obligations as authors under the International Committee of Medical Journal Editors (ICMJE) criteria. Furthermore, clinical study documents (e.g. study report, study protocol, statistical analysis plan) and participant clinical study data are available to be shared after publication of the primary manuscript in a peer-reviewed journal and if regulatory activities are complete and other criteria met per the BI Policy on Transparency and Publication of Clinical Study Data: https://trials.boehringer-ingelheim.com/. Prior to providing access, documents will be examined, and, if necessary, redacted and the data will be de-identified, to protect the personal data of study participants and personnel, and to respect the boundaries of the informed consent of the study participants. Clinical Study Reports and Related Clinical Documents can also be requested via the link https://trials.boehringer-ingelheim.com/. All requests will be governed by a Document Sharing Agreement. Bona fide, qualified scientific and medical researchers may request access to de-identified, analysable participant clinical study data with corresponding documentation describing the structure and content of the datasets. Upon approval, and governed by a Data Sharing Agreement, data are shared in a secured data-access system for a limited period of 1 year, which may be extended upon request. Researchers should use the https://trials.boehringer-ingelheim.com/ link to request access to study data.

## Abbreviations

ALAT: Latin American Thoracic Society
ATS: American Thoracic Society
BMI: Body mass index
ERS: European Respiratory Society
FVC: Forced vital capacity
HRCT: High-resolution computed tomography
IIP: Idiopathic interstitial pneumonia
ILD: Interstitial lung disease
IPF: Idiopathic pulmonary fibrosis
JRS: Japanese Respiratory Society
PFT: Pulmonary function test
SD: Standard deviation
SGRQ: St. George’s Respiratory Questionnaire
