# Supplementary material for: Clinical course of IPF in Italian patients during 12 months of observation: results from the FIBRONET observational study
Source: Respir Res. 2021 Feb 24;22:66. doi: 10.1186/s12931-021-01643-w (PMC7903602; doi:10.1186/s12931-021-01643-w)
Supplement: Supplementary file 1 — Additional file 1. Additional tables and figures. [file 12931_2021_1643_MOESM1_ESM.docx]

**Supplementary material**

**Clinical course of IPF in Italian patients during 12 months of observation: results from the FIBRONET observational study**

Venerino Poletti^1,2^, Carlo Vancheri^3^, Carlo Albera^4^, Sergio Harari^5^, Alberto Pesci^6^, Rosa Metella Refini^7^, Benedetta Campolo^8^, Giovanna Crespi^8^, Sara Rizzoli^9^, on behalf of the FIBRONET study group.

# **Table S1.** Sensitivity analyses: Mean FVC% predicted during 12 months of observation

|  | **N** | **Mean** | **SD** |
| --- | --- | --- | --- |
| **Sensitivity analysis 1** |  |  |  |
| Baseline | 196 | 80.01 | 19.23 |
| 12-month follow-up | 183 | 80.18 | 20.41 |
| **Sensitivity analysis 2** |  |  |  |
| Baseline | 196 | 80.01 | 19.23 |
| 12-month follow-up | 173 | 80.12 | 20.31 |

Sensitivity analysis 1: Imputation of missing FVC% predicted at 12-month follow-up (LOCF applied). Imputation was not performed for patients with only baseline value available.

Sensitivity analysis 2: Imputation of missing FVC% predicted at 12-month follow-up (LOCF applied). Imputation was not performed for patients with only baseline and 3-month follow-up values available.

FVC, forced vital capacity; LOCF, Last Observation Carried Forward; SD, standard deviation.

# Table S2. St. George’s Respiratory Questionnaire scores during the observation period

|  | **N** | **Median  (25^th^ percentile–75^th^ percentile)** |
| --- | --- | --- |
| TOTAL score  Baseline | 181 | 39.82 (23.40–56.49) |
| 6-month follow-up | 137 | 41.14 (24.48–55.79) |
| 12-month follow-up | 134 | 39.60 (23.80–56.77) |
| SYMPTOMS component score  Baseline | 181 | 42.26 (27.29–57.98) |
| 6-month follow-up | 137 | 35.96 (21.97–53.11) |
| 12-month follow-up | 134 | 42.21 (24.72–56.08) |
| ACTIVITIES component score Baseline | 181 | 54.39 (35.80–72.89) |
| 6-month follow-up | 137 | 59.46 (41.48–73.04) |
| 12-month follow-up | 134 | 58.88 (35.47–77.85) |
| IMPACTS ON DAILY LIFE component score  Baseline | 181 | 28.16 (12.26–47.09) |
| 6-month follow-up | 137 | 29.77 (13.04–46.69) |
| 12-month follow-up | 134 | 27.32 (13.50–46.39) |

# Table S3. Adverse events and serious adverse events

|  | **N=209** |
| --- | --- |
| Number of SAEs | 17 |
| Deaths | 13 |
| Deaths due to SAE | 11 |
|  | n (%) |
| Patients with ≥1 AE | 86 (41.1) |
| Patients with ≥1 SAE | 16 (7.7) |

AE, adverse event; SAE, serious adverse event.

# Table S4. Comparison of baseline characteristic outcomes in five national registries

|  | **FIBRONET** | **AIPFR**  **[1]** | **FINNISH**  [2] | **INSIGHT**  [3] | **SWEDISH**  [4] |
| --- | --- | --- | --- | --- | --- |
| Country | Italy | Australia | Finland | Germany | Sweden |
| Patients (n) | 209 | 647 | 111 | 502 | 71 |
| FVC% predicted | 80.0% (±19.2) | 81.0% (21.7) | 80.4% | 67.0% (18.2) | 72.3% (16.9) |

Data are presented as mean (SD).

**References**

1. Jo HE, Glaspole I, Grainge C, Goh N, Hopkins PM, Moodley Y, Reynolds PN, Chapman S, Walters EH, Zappala C, et al: **Baseline characteristics of idiopathic pulmonary fibrosis: analysis from the Australian Idiopathic Pulmonary Fibrosis Registry.** *Eur Respir J* 2017, **49:**1601592.

2. Kaunisto J, Salomaa ER, Hodgson U, Kaarteenaho R, Kankaanranta H, Koli K, Vahlberg T, Myllarniemi M: **Demographics and survival of patients with idiopathic pulmonary fibrosis in the FinnishIPF registry.** *ERJ Open Res* 2019, **5:**00170-02018.

3. Behr J, Kreuter M, Hoeper MM, Wirtz H, Klotsche J, Koschel D, Andreas S, Claussen M, Grohe C, Wilkens H, et al: **Management of patients with idiopathic pulmonary fibrosis in clinical practice: the INSIGHTS-IPF registry.** *Eur Respir J* 2015, **46:**186-196.

4. Ferrara G, Carlson L, Palm A, Einarsson J, Olivesten C, Skold M: **Idiopathic pulmonary fibrosis in Sweden: report from the first year of activity of the Swedish IPF-Registry.** *Eur Clin Respir J* 2016, **3:**31090.

# Supplementary figure legends

# Figure S1. Median SGRQ scores during the observation period

SGRQ, St. George’s Respiratory Questionnaire.

# Figure S2. GP and specialist outpatient visits for IPF, IPF exacerbations or IPF-related adverse events from diagnosis to the end of the observation period

IPF, idiopathic pulmonary fibrosis; GP, general practitioner.
